# Influence of Lactation, Age and Foaling Factors on the Quality Composition, Fatty and Amino Acid Profile of Mare’s Milk Under Pasture Conditions

**DOI:** 10.3390/foods14162880

**Published:** 2025-08-19

**Authors:** Togzhan Boranbayeva, Zhanna Dossimova, Dulat Zhalelov, Aruzhan Zhunisbek, Ayazhan Bolat, Maxat Toishimanov

**Affiliations:** 1 Technology and Food Safety Department, Kazakh National Agrarian Research University, 050010 Almaty, Kazakhstan; togzhan.boranbayeva@kaznaru.edu.kz (T.B.); zhalelov.dulat@kaznaru.edu.kz (D.Z.); 2 Reference Laboratory of Dairy Products, Kazakh National Agrarian Research University, 050010 Almaty, Kazakhstan; zhanna.dossimova@kaznaru.edu.kz (Z.D.); zhunisbek.aruzhan@kaznaru.edu.kz (A.Z.); ayazhan.bolat@kaznaru.edu.kz (A.B.); 3 Food and Environment Safety Laboratory, Kazakh National Agrarian Research University, 050010 Almaty, Kazakhstan

**Keywords:** mare milk, lactation period, fatty and amino acid, atherogenic index, thrombogenic index, Kazakh mare

## Abstract

This study investigated the effects of lactation period, foaling month and number, mare age, and regional factors on the quality parameters, amino acid composition, fatty acid profile, and nutritional indices of Kazakh mare’s milk under pasture conditions. A total of 240 milk samples were collected from Almaty and Zhambyl regions during the summer and autumn lactation periods. Standard physicochemical analyses determined fat, protein, casein, TS, and SNF contents, while amino acids were quantified via HPLC and fatty acids by GC. Significant seasonal differences were observed: summer milk contained higher PUFA (18.29%) and n-3 (5.71%) levels and exhibited lower SFA and AI values, indicating superior nutritional quality. Milk from younger mares (4 to 6 years) showed elevated essential amino acids and better lipid health indices compared to older mares. Zhambyl region samples had higher unsaturated fatty acids and SNF, while Almaty milk exhibited higher SFA and casein content. Amino acid profiling revealed that summer milk was enriched in glutamic acid, aspartic acid, serine, and histidine, whereas autumn milk contained more valine, leucine, methionine, and cysteine. PCA revealed distinct clustering based on season, mare age, and foaling period, confirming their substantial roles in shaping milk composition. These findings highlight that mare age, lactation period, and foaling timing significantly affect the nutritional quality of the mare’s milk. These results provide valuable insights for optimizing milk production and kumys fermentation strategies under traditional pasture-based systems.

## 1. Introduction

Mare’s milk has long held a vital place in the diets of nomadic populations in Central Asia, including Kazakhstan, where it is consumed both in fresh and fermented forms. Traditionally valued for its restorative and therapeutic effects, mare’s milk is increasingly recognized for its unique nutritional profile, which includes a rich composition of bioactive compounds, vitamins, minerals, polyunsaturated fatty acids (PUFAs), and high-quality proteins. Contemporary interest in mare’s milk has expanded from traditional use to its potential application in functional foods, particularly in pediatric and therapeutic nutrition [1,2,3,4].

Kazakh horse breeds are well adapted to local pasture conditions and play a significant role in milk production. Mare milk from these breeds is not only culturally significant but also demonstrates compositional characteristics influenced by genetic background and environmental factors, such as regional forage variability, seasonal, and mare physiology [5,6,7].

Mare’s milk stands out among dairy species due to its digestibility and composition, which more closely resembles human milk than bovine milk. It contains a high proportion of whey proteins, including albumins, globulins, and bioactive peptides, along with fine casein micelles. This contributes to enhanced biological value and digestibility [8,9]. The dominant amino acids include glutamic acid, aspartic acid, and phenylalanine, with significant levels of valine, tyrosine, and methionine, supporting a wide array of physiological functions. Variations in amino acid composition have been linked to lactation stage, breed, and environmental conditions [10,11,12].

Lipids in mare’s milk, while present in lower concentrations compared to ruminant milk, are notable for their high PUFA content and favorable health indices. The fatty acid profile, particularly the ratio of omega-6 to omega-3 (n-6/n-3), as well as the atherogenic index (AI) and thrombogenic index (TI), are widely used indicators of cardiovascular health risk [13,14]. Mare’s milk fat is characterized by elevated levels of essential fatty acids such as linoleic (C18:2n6) and α-linolenic acid (C18:3n3), contributing to anti-inflammatory and immunomodulatory effects. Additionally, the lipid fraction exhibits bactericidal activity, likely due to the PUFA-rich profile, which impairs the growth of pathogenic microflora. Compared to other types of milk, mare’s milk demonstrates lower AI and TI values, suggesting potential cardiovascular protective effects [15,16,17].

The physicochemical composition of mare’s milk is influenced by several interrelated factors, including the stage of lactation, mare age, parity (number of foalings), month of foaling, and regional climatic conditions. These variables collectively affect both major (fat, protein, lactose) and minor components (amino acids, fatty acids, bioactive peptides), consequently altering the milk’s suitability for fresh consumption, fermentation, and therapeutic applications. Importantly, the high lactose content and fine emulsion of milk fat in mare’s milk lead to rapid acidification and spoilage, necessitating timely processing or fermentation to preserve its nutritional value and safety [18,19,20,21,22]. Furthermore, milk composition is significantly modulated by feeding regimes, with pasture-based systems resulting in milk that is richer in PUFAs, antioxidants, and health-promoting compounds compared to confined or concentrate-fed systems [23,24]. In this study, all mare milk samples were collected under extensive pasture conditions, where the animals grazed natural steppe vegetation typical of the southern regions of Kazakhstan (Almaty and Zhambyl). This grazing system reflects traditional Kazakh nomadic practices and plays a critical role in shaping the milk’s bioactive profile.

Despite growing global interest in the nutritional value of mare’s milk, there remains a notable lack of comprehensive data on the compositional dynamics of Kazakh mare’s milk, particularly under standardized pasture-based systems. Prior studies have focused either on general composition or fermentation outcomes but have not integrated key quality indicators—such as fat, protein, casein, lactose, and total solids—with detailed amino acid and fatty acid profiling. Moreover, limited research has evaluated health-related lipid indices such as the AI, TI, and n-6/n-3 ratio in relation to mare age, foaling parameters, and regional variability. This study aims to fill this gap by providing an in-depth analysis of mare’s milk composition, collected under pasture conditions, across multiple biological and environmental factors.

This study aims to address the lack of integrated compositional data for Kazakh mare’s milk by providing a comprehensive analysis of its physicochemical characteristics, amino acid and fatty acid profiles, and lipid health indices (AI, TI, n-6/n-3 ratio) under pasture-based management. Samples were collected across multiple biological (lactation stage, mare age, foaling number) and environmental (seasonal variation) factors to evaluate their combined effects on milk quality and to compare these results with regional and international data.

## 2. Materials and Methods

### 2.1. Study Design and Mare Raw Milk Samples

The study was conducted on a total of 48 Kazakh mares maintained under natural pasture-based conditions, without any supplementary feeding, to reflect traditional nomadic management systems. The animals were housed at two representative farming enterprises: the “Sadygul” enterprise in Asa District, Zhambyl Region (43°07′43″ N 71°04′57″ E; located at 481 m above the sea level), and the “Zhumabayev” enterprise in Karasai District, Almaty Region (43°07′27″ N 76°32′25″ E.; located at 1587 m above sea level)—both located in southern Kazakhstan. Both sites represent distinct agroecological zones—semi-arid steppe and mountain foothills, respectively.

All mares selected for the study were breed-typical Kazakh mares, homogenous in phenotypic type and maintained in good body condition throughout the study. It is important to note that all foalings occurred between April and July, thereby enabling the assessment of mare’s milk composition across distinct stages of lactation under uniform seasonal and management conditions. The foals born to these mares exhibited balanced morphological development and optimal body condition, indicative of healthy maternal nutrition and environmental adaptation. This consistent management and environmental exposure across both sites provides a standardized basis for analyzing regional and physiological influences on the chemical composition and nutritional quality of mare’s milk.

The lactation period of the studied mares lasted for eight months, from April to November. Although milking began in April, milk samples for this study were collected only from July onwards, as early lactation sampling in spring poses potential risks to foal development. During the first month of lactation, the mares were not milked to ensure unrestricted access to milk for the foals.

Milk samples were collected in the first half of the day. Before each milk collection, the teat area was washed with clean water and thoroughly dried. Milking was performed manually. A 1000 mL aliquot was placed into a sterile polypropylene bottle. The samples were immediately stored in a portable refrigerated container and transported to the laboratory on the same day for analysis. The chemical composition of the collected milk samples was analyzed on the day of collection. For fatty acid analysis, milk fat was separated by centrifugation on the same day. For amino acid analysis, a portion of each sample was transferred into vials designated for hydrolysis.

The mares included in this study ranged in age from 4 to 13 years. A total of 48 Kazakh mares were included, equally distributed between two regions—Almaty and Zhambyl—with 24 mares sampled in each region over a 5-month lactation period, resulting in a total of 240 milk samples. The samples were stratified into five experimental factors: (1) Region: Almaty (*n* = 120) and Zhambyl (*n* = 120); (2) Lactation period: summer (July–August; *n* = 96) and autumn (September–November; *n* = 144); (3) Number of foalings: first foaling (*n* = 20), two to four foalings (*n* = 130), and five or more foalings (*n* = 90); (4) Foaling month: April (*n* = 60), May (*n* = 70), June (*n* = 80), and July (*n* = 30); (5) Mare age: 4–6 years (*n* = 80), 7–9 years (*n* = 76), and 10 years or older (*n* = 84).

### 2.2. Pasture Conditions

Throughout the study, the mares were maintained exclusively on natural pastures under traditional extensive grazing systems without any supplementary feeding. The pastures in Asa District (Zhambyl Region; elevation 481 m) are characteristic of dry steppe ecosystems, dominated by drought-tolerant grass species such as *Stipa capillata* (feather grass), *Festuca valesiaca* (sheep fescue), and *Agropyron cristatum* (crested wheatgrass), alongside leguminous forbs including *Astragalus* spp. and *Medicago falcata* (yellow alfalfa).

In contrast, the Karasai District pastures (Almaty Region; elevation 1587 m) reflect a montane-meadow steppe environment, richer in species diversity and biomass due to cooler temperatures and higher rainfall. The predominant species include *Poa pratensis*, *Bromus inermis* (smooth brome), *Trifolium pratense* (red clover), and *Onobrychis arenaria* (sainfoin), as well as aromatic and medicinal herbs such as *Artemisia spp.*, *Achillea millefolium*, and *Thymus serpyllum* [24].

### 2.3. Determination of Physical and Chemical Properties

Immediately after collection, milk samples were placed in a portable refrigerator and transported to the laboratory. Upon arrival, samples were gently mixed to ensure homogeneity and stored at 4 °C until analysis, which was carried out within 24 h. Milk samples were evaluated for the content of fat, protein, casein, lactose, solids-not-fat (SNF), total solids (TS), citric acid, urea, and acidity (°D) contents by infrared spectroscopy using Milko-Scan FT1 analyzer calibrated for mare milk (Foss Electric, Denmark). The Milko-Scan FT1 analyzer requires a minimum of 25 mL of milk for duplicate analysis of each sample.

### 2.4. Fatty Acid Determination

The identification and quantification of fatty acid methyl esters (FAMEs) in mare’s milk were conducted using gas chromatography (GC), following a modified protocol based on Toishimanov et al. [25]. For sample preparation, 500 mL of raw mare’s milk was divided into 15 mL polypropylene centrifuge tubes and centrifuged at 10,000 rpm for 20 min to separate the fat layer at 4 °C. The resulting upper fat phase was carefully collected into a clean 100 mL tube and mixed with 50 mL of *n*-hexane to extract lipids. The solvent was subsequently evaporated to concentrate the lipid extract for methylation.

For transesterification, 2.70 ± 0.01 g of sodium methylate (Sigma-Aldrich, St. Louis, MO, USA) was dissolved in 25 mL of absolute methanol to prepare a methylating reagent. From the lipid extract, 0.10 ± 0.01 mL of oil was placed into a 15 mL Falcon tube, followed by the addition of 2 mL of *n*-hexane and 0.1 mL of the sodium methylate solution. The contents were vortexed for 1 min and left to react at room temperature for 5 min. After the reaction, the mixture was centrifuged at 3000 rpm for 5 min, and 1 mL of the supernatant was collected for chromatographic analysis.

Gas chromatography was performed using a Shimadzu GC-2010 Plus instrument (Shimadzu Corporation, Kyoto, Japan) equipped with a flame ionization detector (FID). Separation was achieved on a CP-Sil 2560 capillary column (100 m × 0.25 mm ID × 0.20 µm film thickness; Agilent Technologies, Santa Clara, CA, USA), designed for high-resolution analysis of FAMEs. Nitrogen (purity ≥ 99%) was used as the carrier gas, supplied by a Parker Domnick Hunter G1110E nitrogen generator (Hauppauge, NY, USA). The gas flow settings were 30 mL/min for hydrogen, 300 mL/min for air, and 30 mL/min for the make-up gas.

The GC injector was maintained at 250 °C and the detector at 260 °C. Injections were made in split mode with a split ratio of 1:40, and a total flow of 95.5 mL/min. The oven temperature program was as follows: initial temperature at 100 °C held for 5 min; ramped at 4 °C/min to 210 °C (held for 8 min); and then increased at 10 °C/min to 240 °C, maintained for 16.5 min. The injection volume was 1.0 µL, and the total run time was 60 min per sample. Identification of individual fatty acids was carried out by comparing retention times with those of a certified 37-component FAME standard mixture (Supelco, Merck, Darmstadt, Germany) [26].

### 2.5. Amino Acid Determination

The quantification of amino acids (AAs) in unfermented mare’s milk was performed using high-performance liquid chromatography (HPLC) following acid hydrolysis and derivatization with phenyl isothiocyanate (PITC). For hydrolysis, 1000 µL of the milk sample was treated with 6 mol/L hydrochloric acid (HCl) and incubated at 110 °C for 13 h in sealed tubes. After hydrolysis, samples were vacuum-dried.

Derivatization was conducted by resuspending the dried hydrolysate in 150 µL of 0.1 mol/L NaOH, 50 µL of deionized water, and 350 µL of PITC derivatization reagent (a mixture of propanol, PITC, and triethylamine in an 8:1:1 v/v/v ratio). The mixture was incubated at room temperature for 30 min. Following the reaction, excess PITC was removed under a gentle nitrogen stream, and the derivatized sample was reconstituted in 1.5 mL of deionized water. Samples were filtered through a 0.45 µm syringe filter prior to injection. A 10 µL volume of each derivatized sample was injected into the HPLC system.

Chromatographic separation was carried out using a Shimadzu Prominence LC-20 HPLC system (Shimadzu, Kyoto, Japan), equipped with a binary pump (LC-20AD), autosampler (SIL-20AC), degasser (DGU-20A5), column oven (CTO-20A), and ultraviolet detector (SPD-20A), all operated via LCSolution software 1.25 SP1 version. A Thermo Hypersil GOLD C18 reversed-phase column (150 mm × 4 mm, 5 µm particle size) was employed for separation.

Two mobile phases were used: mobile phase A consisted of 99% HPLC-grade acetonitrile and 1% acetic acid, while mobile phase B contained 99.9% ultrapure water, 0.1% acetic acid, and 0.1 M sodium acetate. All solvents were filtered through a 0.2 µm membrane and degassed before use. The flow rate was set to 0.8 mL/min, and the total run time for each chromatographic analysis was 43 min. Detection was carried out at 254 nm [27].

Standard solutions for amino acids—including Aspartic acid, Glutamic acid, Serine, Asparagine, Histidine, Arginine, Threonine, Alanine, Proline, Cysteine, Tyrosine, Valine, Methionine, Cystine, Isoleucine, Leucine, Phenylalanine, and Lysine—were prepared using pharmaceutical-grade standards (purity ≥ 99%, Titan Biotech Ltd.). Each standard was initially dissolved in 0.1 M HCl to obtain a concentration of 1 mg/mL, followed by evaporation and derivatization as described above. Calibration curves were generated from serial dilutions at five concentrations: 1, 10, 25, 50, and 100 µg/mL for each amino acid.

### 2.6. Validation of the GC Analysis

The analysis of FAs using the gas chromatography-flame ionization detection method was validated in accordance with the guidelines of the International Council for Harmonisation [28]. The method validation included assessments of linearity and the calibration range for FA quantification.

Linearity was evaluated using a 37-component FAME mix standard, with each FAME component identified based on its retention time and chromatographic profile within the standard mix. The GC conditions, including column temperature, flow rate, and split ratio, were optimized to achieve improved FA separation.

Method precision was assessed through repeatability, which was validated by performing five consecutive analyses using the standard mix solution. The precision of the chromatographic system was further confirmed by determining the relative standard deviation (%RSD) of retention times and peak areas. Additionally, five injections were performed over three consecutive days to verify system reproducibility.

### 2.7. Validation of the HPLC Analysis

The method for the determination of AAs in mare’s milk samples was validated in accordance with the ICH guidelines. Each derivatized AA standard was individually identified based on its retention time, and all AA compounds were subsequently analyzed as a mixture.

To assess linearity, triplicate solutions of derivatized AAs were prepared at final concentrations ranging from 1 to 100 µg/mL. The HPLC conditions for amino acid determination were optimized to enhance separation efficiency by adjusting parameters such as column temperature, flow rate, and mobile phase composition. Method precision was evaluated through triplicate analyses of standard solutions conducted over three consecutive days. Accuracy was determined by calculating the relative standard deviation (%RSD) of peak areas for standard samples at three different concentrations.

### 2.8. Fatty and Amino Acids Ratios

Saturated FA (SFA), unsaturated FA (USFA), monounsaturated FA (MUFA), polyunsaturated FA (PUFA), n-6 and n-3 ratios of mare milk were determined by Reis et al. 2022 [29].

SFA = C4:0 + C6:0 + C8:0 + C10:0 + C12:0 + C14:0 + C16:0 + C17:0 + C18:0 + C20:0.

MUFA = C12:1 + C14:1 + C15:1 + C16:1 + C17:1 + C18:1c9 + C20:1.

PUFA = C18:2 + C18:3n3 + C18:3n6 + C20:2 + C20:3n3 + C20:4n6 + C22:5+ C22:5n3.

USFA = sum of MUFA and PUFA.

Omega-6 fatty acids (n-6) = C18:2n6 + C20:2 + C20:3 + C20:4.

Omega-3 fatty acids (n-3) = C18:3n3 + 20:3n3 + C20:5 + C22:5n3

The atherogenic index (AI) and thrombogenic index (TI) of mare’s milk samples were determined using the following equations [13]:

AI = (C12:0 + 4 × C14:0 + C16:0)/USFA

TI = (C14:0 + C16:0 + C18:0)/[(0.5 × MUFA) + (3 × n-3) + (0.5 × n-6) + (n-3/n-6)]

Essential amino acids (EAA) and non-essential amino acids (NEAA) were calculated by Purkiewicz et al. [30].

EAA = His + Thr + Val + Met + Ile + Leu + Phe + Lys

NEEA = Asp + Glu + Ser + Asn + Arg + Ala + Pro + Cys + Tyr

### 2.9. Statistical Analysis

All statistical analyses were performed using JMP Pro 16.0 (SAS Institute Inc., Cary, NC, USA). The data were subjected to multifactorial analysis of variance (ANOVA) model to evaluate the effects of five categorical factors—region (Almaty and Zhambyl), lactation period (summer and autumn), number of foalings (1, 2 to 4, ≥5), foaling month (April to July), and mare age (4 to 6, 7 to 9, and ≥10 years)—on the chemical composition, fatty acid profiles, and amino acid concentrations of Kazakh mare’s milk. To determine the relative contribution of each factor to the variation in each biochemical parameter, *p*-values were extracted for each main effect and summarized in the corresponding results tables. Results are expressed as mean ± standard deviation (SD). Significance levels are reported as *p*-values, with different superscript letters in tables indicating statistically significant differences among group means at *p* < 0.05 according to Tukey’s HSD test.

Principal component analysis (PCA) was performed to evaluate correlations among the parameters studied. A general linear model was constructed with milk quality content as the dependent variable, and region, mare age, and foaling number as fixed effects. Interaction terms between region and both mare age and foaling number were included.

## 3. Results

### 3.1. Fatty Acid Determination Method Validation

A comprehensive quantitative assessment of FAs was conducted, with calibration curves established over a range from 20.2 µg/mL to 612 µg/mL. These curves were derived from five distinct concentrations. As presented in Table 1, the validation of FA components included retention times, correlation coefficients, linear equations, analytical ranges, as well as limits of detection (LOD) and quantification (LOQ). The correlation coefficients exceeded 98%, demonstrating the high performance of the detector.

**Figure 1:**
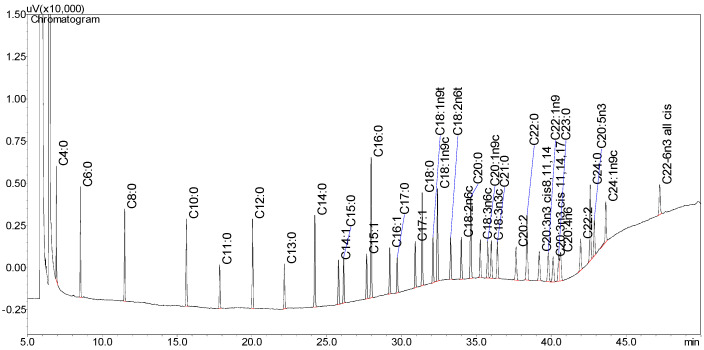
GC chromatogram of 37 FAME mix standard. Analyzed on CP-Sil 2560 high-polarity capillary column (100 m × 0.250 mm × 0.25 µm). Column temperature was programmed as follows: 100 °C (5 min) increased 4 °C/min, 210 °C (8 min) at 2 °C/min, and increased by 10 °C/min to 240 °C 240 °C (16.5 min). The *x*-axis represents the time, and the *y*-axis represents response intensity.

The accuracy and precision of the method were confirmed through repeated analyses of the FAME Mix standard, with retention times illustrated in Figure 1. The repeatability, expressed as the percentage relative standard deviation (%RSD) for retention times, did not surpass 0.5%. Similarly, the precision for peak areas remained below 1.0%, and for retention times, it was under 0.3% in identical conditions. The LOD ranged from 0.29 µg/mL to 1.95 µg/mL, while the LOQ varied between 2.06 µg/mL and 3.95 µg/mL, indicating the high sensitivity of the method. Based on these results, the analytical method is considered appropriate for the identification of fatty acids (FAs) in mare’s milk, in accordance with the ICH guidelines (2005).

### 3.2. Amino Acid Determination Method Validation

**Figure 2:**
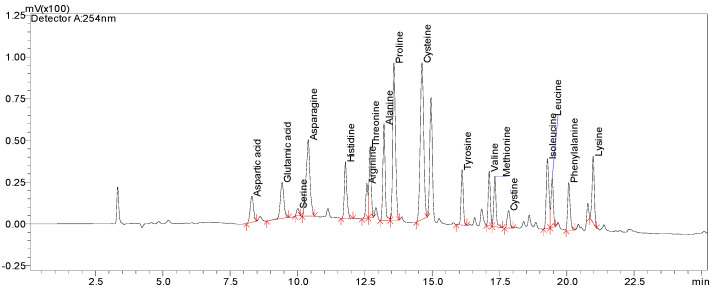
HPLC chromatogram of a mixture of amino acids. Analyzed on a Thermo Hypersil GOLD C18 HPLC column (150 mm × 4 mm, 5 µm), the UV detection was at 254 nm. The *x*-axis represents the time, and the *y*-axis represents response intensity.

Calibration curves for all analyzed AAs were constructed using standard solutions within the concentration range of 1–100 µg/mL. As presented in Table 2, the peak area of each AA standard was plotted against its concentration, and linearity parameters were determined through linear regression analysis. The coefficient of determination (R^2^) values were ≥ 0.995 for all AA compounds, indicating a high degree of linearity. The LOD and LOQ were assessed through progressive dilution of standard solutions. The obtained LOD values ranged from 0.03 µg/mL to 0.16 µg/mL, while LOQ values varied between 0.10 µg/mL and 0.47 µg/mL for all analyzed AAs, demonstrating the method’s high sensitivity. The AA method’s accuracy and precision were validated through repeated analysis of the prepared amino acid mix standard, whose retention times and peaks are shown in Figure 2.

### 3.3. Quality Compositions

Table 3 presents the chemical composition of mare’s milk from the Almaty and Zhambyl regions during different lactation periods and according to various biological factors.

The fat content of Kazakh mare’s milk was significantly higher in the Zhambyl region (1.29 ± 0.37%) compared to the Almaty region (0.85 ± 0.39%) (*p* < 0.0001). A strong seasonal effect was also observed, with autumn samples exhibiting markedly higher fat content (1.31 ± 0.38%) than those collected in summer (0.71 ± 0.27%) (*p* < 0.0001). The foaling month had a statistically significant influence on fat levels (*p* = 0.0160), with the highest fat concentrations found in milk from mares that foaled in July (1.36 ± 0.15%). In contrast, neither the number of foalings (*p* = 0.4781) nor mare age (*p* = 0.6632) significantly affected milk fat content.

Protein content was significantly higher in the Almaty region (1.71 ± 0.18%) than in the Zhambyl region (1.58 ± 0.33%) (*p* < 0.0001). No significant variation in protein levels was detected by the lactation season (*p* = 0.2535), number of foalings (*p* = 0.5717), or mare age (*p* = 0.2790). However, the foaling month showed a mild but significant effect (*p* = 0.0453), with April-foaling mares producing milk with the highest protein content (1.68 ± 0.13%).

A highly significant seasonal effect was observed for casein content, with summer samples showing higher concentrations (1.26 ± 0.14%) compared to autumn samples (1.04 ± 0.18%) (*p* < 0.0001). Additionally, casein content varied by foaling month (*p* = 0.0294), with the highest values found in milk from mares that foaled in April and May. No significant differences in casein content were detected between regions (*p* = 0.8648), across foaling numbers (*p* = 0.6519), or mare age groups (*p* = 0.4478).

Lactose concentration remained stable across all groups, with no statistically significant differences detected between regions (*p* = 0.4979), lactation months (*p* = 0.1588), the foaling numbers (*p* = 0.7136), the foaling months (*p* = 0.1302), or mare age groups (*p* = 0.7905). Average lactose content ranged between 6.41% and 6.51%.

Milk urea levels were significantly higher in samples from the Almaty region (46.39 ± 3.51 mg/dL) than in those from Zhambyl (31.64 ± 1.96 mg/dL) (*p* = 0.0004). A seasonal difference was also observed (*p* = 0.0106), with higher values detected in autumn. However, no statistically significant differences were associated with the foaling number (*p* = 0.7848), the foaling month (*p* = 0.2291), or mare age (*p* = 0.6087).

Citric acid levels were significantly higher in the Zhambyl region (0.10 ± 0.02%) than in the Almaty region (0.05 ± 0.01%) (*p* = 0.0003). No significant differences were found across lactation seasons (*p* = 0.8023), the foaling numbers (*p* = 0.6016), foaling months (*p* = 0.8090), or mare age categories (*p* = 0.0886).

A strong seasonal effect was recorded for milk acidity, with summer samples demonstrating significantly higher values (10.24 ± 0.39°D) compared to autumn (6.82 ± 0.07°D) (*p* < 0.0001). No significant effects were found for region (*p* = 0.1177), foaling number (*p* = 0.6386), foaling month (*p* = 0.2562), or mare age (*p* = 0.6710).

SNF content was significantly influenced by both region (*p* = 0.0114) and lactation season (*p* = 0.0008). Higher SNF values were observed in the Zhambyl region and during the autumn period. There were no significant effects related to foaling number (*p* = 0.6871), foaling month (*p* = 0.3723), or mare age (*p* = 0.8881).

Total solids content differed significantly by lactation season (*p* = 0.0447), with higher concentrations observed in autumn (9.19 ± 1.06%) compared to summer (8.86 ± 0.47%). No significant differences in TS were observed for region (*p* = 0.7089), foaling number (*p* = 0.8693), foaling month (*p* = 0.1114), or mare age (*p* = 0.3560).

### 3.4. Fatty Acid Composition of Kazakh Mare’s Milk According to Region, Lactation Period, and Biological Factors

The concentration of short-chain fatty acids, particularly butyric acid (C4:0) and caproic acid (C6:0), showed strong dependence on the lactation period. In autumn, C4:0 content was significantly higher (0.21 ± 0.03%) compared to summer (0.04 ± 0.01%) (*p* = 0.0004), indicating enhanced microbial fermentation or fat mobilization in later lactation. C6:0 also followed this trend, decreasing from 0.18 ± 0.01% in summer to 0.07 ± 0.01% in autumn (*p* = 0.0001). A statistically significant effect of foaling month on C4:0 was observed (*p* = 0.0130), with the July-foaling mares exhibiting the highest levels (0.29 ± 0.14%). However, region, foaling number, and mare age had no significant influence on SCFA levels (*p* > 0.25 for all), as shown in Table 4.

Among medium-chain saturated fatty acids, notable regional differences were recorded. The concentration of caprylic acid (C8:0) was significantly higher in Almaty milk (2.32 ± 0.80%) compared to Zhambyl (1.88 ± 0.81%) (*p* = 0.0079). Capric acid (C10:0) showed the strongest regional effect, with levels dropping from 6.18 ± 1.66% in Almaty to 4.46 ± 1.11% in Zhambyl (*p* < 0.0001), and also differed significantly by lactation season (*p* = 0.0462) and foaling month (*p* = 0.0056), being higher in spring-foaling mares.

Palmitic acid (C16:0), the most abundant saturated fatty acid, was significantly influenced by lactation month (*p* = 0.0001), foaling month (*p* = 0.0031), and mare age (*p* = 0.0359). Summer milk contained higher C16:0 (24.02 ± 3.25%) compared to autumn (20.84 ± 3.65%). Younger mares (4–6 years) had slightly higher palmitic acid levels than older age groups. Stearic acid (C18:0) was strongly affected by lactation month (*p* = 0.0001), with higher levels in summer (1.63 ± 0.16%) versus autumn (0.62 ± 0.12%). It also varied by foaling number (*p* = 0.0249) and foaling month (*p* = 0.0450), though no regional or age-related differences were observed.

Palmitoleic acid (C16:1) concentrations were significantly higher in the Zhambyl region (8.59 ± 1.98%) compared to Almaty (6.55 ± 1.99%) (*p* < 0.0001). This suggests possible regional differences in lipid metabolism or feeding practices. Heptadecenoic acid (C17:1) showed significant effects for region (*p* = 0.0010) and season (*p* = 0.0172), being elevated in autumn.

Oleic acid (C18:1n9c) levels were also higher in Zhambyl (20.96 ± 4.84%) than in Almaty (18.28 ± 3.79%) (*p* = 0.0022). Additionally, foaling number (*p* = 0.0472) and mare age (*p* = 0.0378) were influential, with younger mares and those with 2–4 foalings tending to produce milk with higher MUFA content.

PUFAs showed the most substantial and widespread variability. Linoleic acid (C18:2n6c) was significantly higher in Almaty (17.53 ± 4.37%) than in Zhambyl (12.27 ± 4.32%) (*p* < 0.0001) and was influenced by mare age (*p* = 0.0321). α-Linolenic acid (C18:3n3c) was higher in summer (5.71 ± 0.47%) than in autumn (1.27 ± 0.22%) (*p* < 0.0001), and was also significantly influenced by region (*p* = 0.0220), foaling number (*p* = 0.0484), foaling month (*p* = 0.0306), and mare age (*p* = 0.0338).

Likewise, γ-linolenic acid (C18:3n6c) exhibited substantial differences by region, lactation period, foaling number, and mare age (all *p* < 0.05), with values increasing sharply from 0.87 ± 0.14% in Almaty to 2.59 ± 0.43% in Zhambyl. This suggests that regional and physiological factors may strongly influence long-chain PUFA synthesis or transfer into milk.

### 3.5. Influence of Region, Season, and Physiological Factors on Fatty Acid Composition and Health Indices

Milk from mares in Almaty displayed a higher PUFA content (36.94%) compared to Zhambyl (29.92%), indicating a potentially more favorable fatty acid profile for human health. Conversely, Zhambyl samples had higher MUFA levels (30.95%) than Almaty (26.26%), largely due to elevated oleic acid levels (C18:1n9c). The n-3 content was markedly higher in Zhambyl (6.36%) than in Almaty (3.24%), contributing to a lower and more desirable n-6/n-3 ratio (2.34 in Zhambyl vs. 5.41 in Almaty). Accordingly, Zhambyl milk exhibited improved cardiovascular health indices, with lower AI (1.14) and TI (0.78) values compared to Almaty (1.33 and 0.84, respectively).

Fatty acid profiles varied substantially between the summer and autumn lactation periods. Summer milk was characterized by a notably higher PUFA content (37.79%), primarily due to enhanced omega-3 (7.56%) and omega-6 levels (29.10%). In contrast, autumn milk exhibited elevated SFA (55.26%) and MUFA (29.62%), with reduced PUFA (21.85%). These seasonal changes resulted in a superior lipid profile in summer, as indicated by a more favorable n-6/n-3 ratio (3.85 vs. 17.51), and lower AI (1.26) and TI (0.84) values compared to autumn (1.23 and 0.71, respectively). This seasonal variation suggests that pasture-based summer feeding promotes the biosynthesis or transfer of beneficial unsaturated fatty acids into the milk.

Mares with 5 or more foalings produced milk with the lowest SFA (46.25%) and highest PUFA content (34.61%), particularly omega-3 (5.56%), resulting in a remarkably low n-6/n-3 ratio (1.76). These animals also exhibited the most favorable cardiovascular indices (AI = 1.19, TI = 0.81). In contrast, first-parity mares had the highest SFA (58.13%) and lowest PUFA (19.68%) levels, with a correspondingly poor n-6/n-3 ratio (7.88). These findings highlight the potential role of maternal physiological maturity and repeated lactation cycles in improving milk quality.

Milk from mares foaling in July demonstrated the most favorable fatty acid profile: the lowest SFA (45.60%), highest PUFA (35.78%), and a balanced omega-6 (25.74%) to omega-3 (5.28%) ratio (4.88). These values coincided with the lowest AI (1.06) and TI (0.72), suggesting improved nutritional and health-promoting properties in late-season milk. In contrast, milk from April foalings had higher SFA (57.31%), lower PUFA (21.87%), and a significantly elevated n-6/n-3 ratio (9.02), resulting in higher AI (1.29) and TI (0.85) indices. These trends may be associated with the progressive adaptation to fresh forage and longer grazing periods in later months.

Mares aged 10 years or older produced milk with the lowest SFA (48.31%) and highest PUFA (32.79%), driven by higher omega-3 levels (4.82%) and reduced n-6/n-3 ratio (3.00). In comparison, younger mares (4–6 years) had higher SFA (55.23%), lower PUFA (22.31%), and a less favorable n-6/n-3 ratio (6.18). The AI and TI indices improved progressively with age, indicating that older mares produce milk with better cardiovascular benefits. This may reflect improved metabolic stability or feed utilization efficiency with advancing age.

Milk from the Zhambyl region contained higher levels of MUFA (30.73%) and PUFA (21.36%), lower SFA (45.60%), and more favorable cardiovascular indices (AI = 1.14; TI = 0.78) compared to the Almaty region. Summer milk showed superior nutritional quality with elevated PUFA (26.86%), n-3 fatty acids (5.71%), and a reduced n-6/n-3 ratio (2.70), while autumn samples were comparatively lower in essential fatty acids. Multiparous mares (≥5 foalings) and those foaling in July produced milk with the highest PUFA (29.58% and 35.78%, respectively), improved n-3 levels, and the most favorable health indices (AI = 1.06; TI = 0.72). Older mares (≥10 years) also yielded milk with enhanced PUFA (26.90%) and reduced SFA (48.48%) compared to younger counterparts. These patterns emphasize that milk quality improves with optimal lactation timing, advanced mare age, and extended reproductive history, especially under pasture-based summer conditions in the Zhambyl region.

### 3.6. Amino Acid Composition of Kazakh Mare’s Milk According to Region, Lactation Period, and Biological Factors

The concentration of aspartic acid in mare’s milk ranged from 0.78 ± 0.12 mg/mL in mares with a single foaling to 2.15 ± 0.16 mg/mL in mares with ≥5 foalings (Table 5). Statistically significant differences were found for lactation period (*p* = 0.0210), foaling number (*p* = 0.0484), foaling month (*p* = 0.0361), and mare age (*p* = 0.0165). The content was higher in autumn (1.82 ± 0.11 mg/mL) than in summer (1.49 ± 0.11 mg/mL), reflecting increased protein synthesis or reduced dilution from milk volume in later lactation. Aspartic acid levels were particularly elevated in mares aged ≥10 years (2.16 ± 0.16 mg/mL), likely associated with higher mammary secretory maturity and metabolic adaptation in older animals.

Glutamic acid was the most abundant amino acid identified, with values ranging from 3.06 ± 0.35 in Almaty to 4.85 ± 0.40 mg/mL in Zhambyl region milk. Statistically significant differences were recorded for region (*p* = 0.0103), lactation period (*p* < 0.0001), foaling number (*p* = 0.0125), and mare age (*p* = 0.0316). Glutamic acid sharply increased in autumn (5.58 ± 0.36 mg/mL), consistent with the concentration effect of reduced milk yield and possible upregulation of amino acid transporters. Parity also played a role: multiparous mares (≥5 foalings) showed consistently higher levels, suggesting enhanced synthetic capacity or longer lactation experience.

Serine levels differed significantly by region (*p* = 0.0334), foaling month (*p* = 0.0296), and mare age (*p* = 0.0234). Milk from Zhambyl showed elevated serine (1.26 ± 0.17 mg/mL), while younger mares (4–6 years) had lower levels (0.64 ± 0.06 mg/mL) compared to mares 7–9 years (1.34 ± 0.17 mg/mL). April-foaling mares had particularly low values (0.63 ± 0.05 mg/mL), potentially linked to the early lactation phase coinciding with limited pasture intake or hormonal factors.

Although asparagine showed no regional or biological variation (all *p* > 0.35), a highly significant seasonal effect was noted (*p* < 0.0001). In autumn, its level increased more than sixfold (0.37 ± 0.04 mg/mL) compared to summer (0.06 ± 0.01 mg/mL), indicating a possible metabolic shift toward nitrogen conservation or increased enzymatic conversion from aspartate under declining milk volume.

Histidine levels were significantly affected by lactation month only (*p* < 0.0001), increasing from 0.12 ± 0.01 mg/mL in summer to 0.35 ± 0.01 mg/mL in autumn. This may reflect its importance in hemoglobin synthesis, immune response, and buffering capacity during metabolic adjustments in late lactation.

Arginine content was also seasonally influenced (*p* < 0.0001), with values nearly doubling from 0.95 ± 0.14 mg/mL in summer to 1.89 ± 0.09 mg/mL in autumn. A significant effect of foaling month was observed (*p* = 0.0112), with April-foaling mares showing the highest levels (1.31 ± 0.12 mg/mL). These findings may reflect arginine’s role in nitric oxide metabolism and lactational vasodilation.

Threonine demonstrated sensitivity to all five factors, including region (*p* = 0.0350), lactation period (*p* = 0.0074), foaling number (*p* = 0.0496), foaling month (*p* = 0.0019), and mare age (*p* = 0.0150). Its concentration peaked in milk from July-foaling mares (1.11 ± 0.38 mg/mL) and older mares (≥10 years), suggesting a regulatory link to mucin production or protein turnover.

Alanine levels were significantly higher in summer (0.22 ± 0.01 mg/mL) than in autumn (0.04 ± 0.00 mg/mL) (*p* < 0.0001), suggesting seasonal metabolic shifts. A mild effect of foaling month was noted (*p* = 0.0469), though region, parity, and age showed no significant differences.

Proline, a key component of collagen, varied significantly only by lactation period (*p* < 0.0001), with higher levels in summer (0.58 ± 0.03 mg/mL) versus autumn (0.28 ± 0.02 mg/mL). The decline may be attributed to changes in connective tissue remodeling or mammary cell turnover rates.

Both sulfur-containing amino acids were affected by season (*p* < 0.0001), with concentrations higher in summer. Cystine also showed significant effects of foaling number (*p* = 0.0476) and foaling month (*p* = 0.0248), indicating enhanced thiol metabolism or redox buffering demands during early lactation.

Tyrosine differed significantly only by lactation month (*p* < 0.0001), increasing from 0.04 ± 0.00 in summer to 0.16 ± 0.01 mg/mL in autumn. This trend may support its role as a precursor for catecholamines during energy metabolism in late lactation.

Valine showed significant seasonal variation (*p* < 0.0001), increasing from 0.76 ± 0.01 mg/mL in summer to 2.36 ± 0.12 mg/mL in autumn. This may be utilized more during energy-demanding phases, and its accumulation could reflect reduced oxidative use in later lactation.

Methionine was affected by region (*p* = 0.0422), lactation period (*p* < 0.0001), and foaling month (*p* = 0.0328). The highest levels were found in Zhambyl (0.15 ± 0.01 mg/mL), with a strong decrease in autumn (0.05 ± 0.00 mg/mL), consistent with shifts in methylation and antioxidant demands.

Isoleucine showed significant effects across all five factors: region (*p* = 0.0262), lactation (*p* < 0.0001), foaling number (*p* = 0.0472), foaling month (*p* = 0.0150), and mare age (*p* = 0.0291). It ranged from 0.49 ± 0.06 in early autumn to 2.73 ± 0.21 mg/mL in late summer, indicating complex regulations linked to muscle protein turnover and energy status.

Leucine levels were relatively stable across all groups except lactation month (*p* < 0.0001). Seasonal effects indicate its regulation may mirror valine and isoleucine due to shared metabolic pathways.

Phenylalanine was influenced significantly only by the lactation period (*p* < 0.0001), increasing during autumn. The absence of other effects suggests stable regulation of this aromatic amino acid under varying physiological states.

Lysine was affected by region (*p* = 0.0152), lactation (*p* < 0.0001), foaling month (*p* = 0.0153), and mare age (*p* = 0.0251), with higher concentrations in autumn, older mares, and in samples from the Zhambyl region. Lysine plays a central role in protein synthesis and immune function and may be particularly sensitive to environmental and physiological conditions.

### 3.7. Total Essential and Non-Essential Amino Acid Content in Mare’s Milk

Mare’s milk from the Zhambyl region contained significantly higher levels of both essential AAs (6.63 mg/mL) and non-essential AAs (11.05 mg/mL) compared to samples from the Almaty region (5.61 and 7.95 mg/mL, respectively). This regional difference may be attributed to differences in pasture botanical composition, climatic factors, and grazing patterns, as the Zhambyl region provides more stable and protein-rich forage during the lactation season. The higher content of both AA categories suggests that milk from this region may be more suitable for developing functional dairy beverages.

A pronounced seasonal trend was observed. Milk collected during the autumn season showed elevated concentrations of essential AAs (7.81 mg/mL) and non-essential AAs (11.88 mg/mL), compared to samples from summer (4.60 and 6.55 mg/mL, respectively). The late-lactation enrichment of amino acids may reflect metabolic adaptations in the mammary gland to meet the nutritional demands of growing foals or to compensate for decreasing milk volume with higher quality. This trend aligns with findings in ruminant species, where late-lactation milk becomes more protein-dense. For product formulation, autumn milk may be superior in both nutritive value and bioactivity.

Foaling number significantly impacted amino acid content. Mares with ≥5 foalings produced milk with the highest essential AA content (6.41 mg/mL) and non-essential AAs (10.40 mg/mL). In contrast, primiparous mares (first-time foaling) had the lowest values (4.37 and 6.82 mg/mL, respectively). This progressive increase with parity may be due to improved endocrine regulation, greater physiological maturity of the mammary tissue, and more efficient nutrient mobilization in multiparous mares. From a breeding and production standpoint, selecting multiparous mares for kumys production may enhance amino acid richness.

The month of foaling also played a critical role. Amino acid contents increased consistently from April to July, with essential AAs rising from 4.24 to 7.10 mg/mL, and non-essential AAs from 7.92 to 10.19 mg/mL. These changes likely reflect seasonal improvements in pasture quality, as summer grasses and legumes are more abundant in amino acids such as lysine, methionine, and glutamine precursors. Additionally, longer grazing durations and higher daylight hours in later months may stimulate metabolic and hormonal factors associated with enhanced milk biosynthesis. Thus, milk obtained in July could be particularly advantageous for nutraceutical applications.

Older mares (≥10 years) produced milk with the highest essential AA content (7.31 mg/mL) and non-essential AAs (11.41 mg/mL), followed by mares aged 7 to 9 years (5.73 and 9.48 mg/mL) and 4 to 6 years (4.61 and 8.12 mg/mL). The age-related elevation may be due to more fully developed secretory epithelial cells and prolonged adaptation of metabolic pathways involved in milk synthesis. While younger mares are often preferred for ease of management, older mares might offer superior milk amino acid profiles, suggesting an important trade-off in herd optimization for quality vs. yield.

### 3.8. Principal Component Analysis of Mare Milk

#### 3.8.1. Principal Component Analysis Results of Quality Parameters in Mare’s Milk

**Figure 3:**
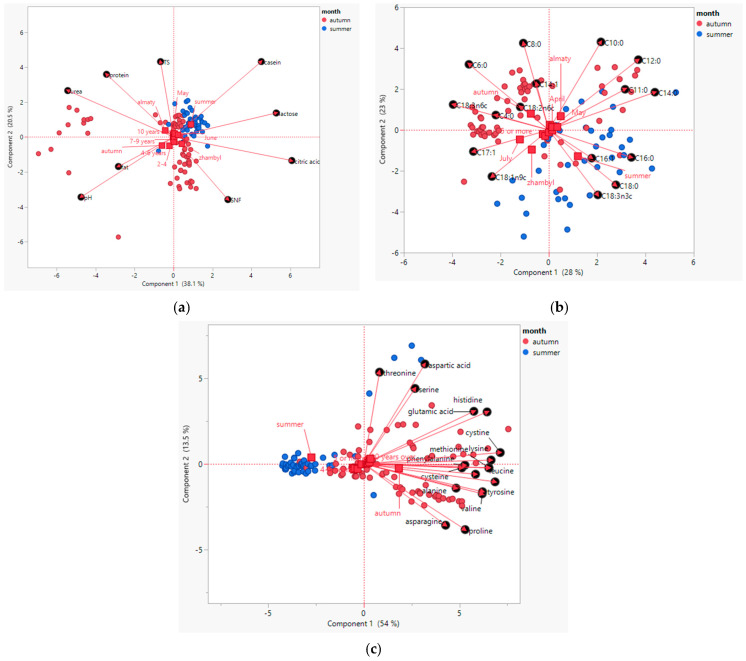
Principal component analysis biplots of mare milk composition by (**a**) quality composition, (**b**) fatty acids, and (**c**) amino acids.

PCA was performed to explore multivariate patterns in the chemical composition of Kazakh mare’s milk in relation to environmental and biological factors (Figure 3a). The first two principal components explained a cumulative 58.6% of the total variance, with PC1 accounting for 38.1% and PC2 for 20.5%, indicating a moderately strong dimensional reduction of the dataset.

The biplot revealed a clear discrimination between milk samples collected during summer and autumn. Samples collected in summer clustered predominantly on the positive side of PC1, strongly associated with higher concentrations of casein, lactose, citric acid, and SNF. These traits are indicative of improved nutritional and technological quality, commonly favored in fermented dairy production such as kumys. Conversely, autumn samples aligned with higher fat, protein, TS, urea, and elevated pH, suggesting a concentration effect likely influenced by reduced water intake or environmental stress conditions during late lactation.

Regionally, milk from Zhambyl aligned closely with summer traits, whereas Almaty was more associated with autumn variables. Notably, foaling-related variables also exhibited directional alignment: milk from mares foaling in June and July, of older age (≥10 years), and with higher parity (≥5 foalings), aligned with summer-related quality markers such as increased lactose and casein. In contrast, milk from mares with 2–4 foalings, younger age (4–6 years), or foaling in April–May clustered with autumn-associated traits.

In summary, PCA underscores that seasonal variation, geographical region, and reproductive parameters (age, parity, and foaling month) collectively structure the variability in mare’s milk composition.

#### 3.8.2. Principal Component Analysis of Fatty Acid Profiles in Mare’s Milk

PCA was conducted to identify patterns in the fatty acid composition of Kazakh mare’s milk in relation to season, region, foaling month, and foaling number. The first two principal components explained a combined 51% of the total variance in the fatty acid dataset, with PC1 accounting for 28% and PC2 for 23%. This indicates a meaningful dimensional reduction, sufficient to capture the primary variation across samples.

The biplot revealed distinct seasonal clustering: autumn samples were primarily distributed along the negative axis of PC1 and the positive axis of PC2, while summer samples were dispersed in the opposite direction. Autumn milk was closely associated with higher levels of short- and medium-chain saturated fatty acids, including C6:0, C8:0, C10:0, and C12:0, as well as monounsaturated C17:1 and pro-inflammatory omega-6 fatty acids such as C18:2n6c and C18:3n6c. These associations suggest a lipid profile richer in saturated and omega-6 fatty acids during autumn, possibly due to changes in forage quality and environmental stress in late lactation.

In contrast, summer milk was associated with higher concentrations of long-chain fatty acids, particularly C14:0, C16:0, C16:1, C18:0, C18:1n9c, and omega-3 fatty acids such as C18:3n3c, which align with improved nutritional and anti-inflammatory properties. These differences likely reflect fresh pasture availability and more favorable metabolic conditions during early lactation in summer.

Regional and reproductive effects were also apparent. Milk from the Zhambyl region, as well as from mares that foaled in June and July, and those with ≥5 foalings, was positively associated with omega-3-rich fatty acids and unsaturated profiles. Conversely, milk from Almaty, mares foaling in April and May, or with fewer foalings, exhibited a profile dominated by saturated and omega-6 fatty acids.

Overall, the PCA confirms that seasonality, geographical origin, and mare-specific factors such as foaling month and parity significantly influence the fatty acid composition of mare’s milk.

#### 3.8.3. Principal Component Analysis of Amino Acid Composition in Mare’s Milk

PCA was applied to the amino acid profile of mare’s milk to elucidate underlying patterns associated with season, mare age, and lactation-related variables. The first two principal components explained a total of 67.5% of the variance, with PC1 accounting for 54% and PC2 for 13.5%. This dimensional reduction effectively captured the key variability in amino acid distribution among samples.

The PCA biplot revealed clear seasonal differentiation: summer samples clustered distinctly on the negative side of PC1, while autumn samples were distributed across the positive side of PC1 and along PC2. Summer milk was associated with elevated concentrations of aspartic acid, threonine, serine, glutamic acid, and histidine, reflecting enhanced levels of polar and acidic amino acids, which are essential for protein synthesis and nitrogen metabolism. This may be attributed to improved pasture quality and higher metabolic activity during early lactation.

In contrast, autumn milk was enriched in branched-chain and sulfur-containing amino acids such as valine, leucine, isoleucine, methionine, cystine, and cysteine, along with glycine, proline, alanine, tyrosine, and asparagine. These amino acids are typically associated with tissue turnover, antioxidant function, and milk protein stability, suggesting a compositional shift linked to late lactation physiology and dietary changes during pasture senescence.

Further, the PCA identified contributions from mare-specific variables. Milk from mares ≥10 years of age, or those in the autumn season, and foaling in earlier months (April–May), clustered closer to the autumn-associated amino acids, indicating a tendency toward a protein profile favoring milk preservation and adaptation to nutritional stress. Conversely, younger mares (4–6 years) and summer lactation aligned with higher levels of aspartic and glutamic acids.

In summary, the PCA revealed that season, mare age, and foaling month significantly influence the amino acid composition of the mare’s milk. These results underscore the biological complexity of milk synthesis and its dependence on both physiological state and environmental context, with implications for kumys production and nutritional evaluation.

## 4. Discussion

The present study provides new insights into how geographical region, mare age, and foaling number influence the compositional quality of mare’s milk. The observed variation in the physicochemical composition of mare’s milk between the Almaty and Zhambyl regions can be attributed to climatic and topographic differences. Milk from the Almaty region exhibited significantly higher fat and protein levels, possibly due to higher elevation pastures (1587 m vs. 481 m), which are known to increase metabolic activity and nutrient density in forage. Our results revealed that both environmental and physiological factors significantly affected milk protein content and possibly other milk quality parameters. Previous research has also shown that the regional origin of animals contributes significantly to milk macrocomponent profiles due to variability in pasture biodiversity and microclimate [31,32,33,34]. Our results align with earlier work by Barłowska et al. [16], who demonstrated that milk composition in cold-blooded mares was sensitive to regional and management-related conditions. Similarly, a recent study by Boranbayeva et al. [35] on Kazakh kumys production reported that the Zhambyl region yielded samples with significantly higher total solids and microbiological quality, supporting the present observation that regional environmental factors—such as pasture type, climate, and altitude—are key drivers of milk quality.

Our findings align with Barłowska et al. [16], who noted that the fat content in cold-blooded Sokólski mares ranged between 1.3% and 1.8%, which is slightly higher than that reported for donkey milk (1.5–1.8%) [36]. Similarly, G Czyżak-Runowska [20] reported comparable fat levels in Polish mare milk. These relatively low-fat values underscore the digestibility and dietary suitability of mare’s milk for individuals with lipid metabolism issues.

Protein content in this study averaged from 1.58% to 1.71%, which is lower than earlier findings on Kazakh and Mongolian breeds, where protein levels typically average 2.2% [37]. Our results are also comparable to those reported by Malacarne et al. [38] in Italian Haflinger mares and support the role of mare’s milk as a potential protein source in functional dairy products. Pieszka et al. examined the chemical composition of mare’s milk from different breeds, including Polish Coldblood, Malopolski, Hucul, and Arabian mares. A key focus was the protein concentration, which is a critical determinant of the nutritional and technological value of mare’s milk. The results revealed statistically significant differences in protein levels among the breeds studied. The average total protein content ranged from 1.80% to 2.30%, with the Hucul and Malopolski mares exhibiting higher protein concentrations compared to Arabian and Polish Coldblood mares. The variation in protein content was attributed to genetic differences, physiological status, and nutritional management of the animals [39]. The significant regional variation in protein levels, with higher values in higher-altitude areas, may be associated with differences in forage quality and animal metabolic responses to environmental stress [40,41].

According to Park et al. [42], mare’s milk typically contains 6.2–7.0% lactose, which is higher than that found in cow’s milk and closely resembles the lactose concentration in human milk. In our study, the lactose levels ranged from 6.3% to 6.8%, depending on the mare’s age, lactation period, and foaling number. This high lactose content supports the mildly sweet taste of mare’s milk and its rapid spoilage rate, due to the fermentation of lactose under natural conditions.

Casein levels, varying from 0.92% to 1.07%, were lower than in bovine milk but are typical for equine milk. The casein content of Kazakh mare’s milk in the present study ranged between 0.45% and 0.72%, which is consistent with the known biochemical characteristics of equine milk. The observed variation in casein levels was influenced by factors such as lactation stage, mare age, and number of foalings, which aligns with the patterns reported in recent investigations of Kazakh mares’ milk under natural pasture conditions [43]. Mare’s milk is notably lower in casein compared to ruminant milk, contributing to its higher digestibility and its suitability for pediatric and therapeutic nutrition [44].

In Central Asia, mare’s milk has long been used for fermented drinks like kumis (also called airag in Mongolia). Studies from Mongolia and neighboring regions consistently highlight the unique composition of mare’s milk—notably low fat and protein but high lactose content. For instance, classic analyses report mare’s milk containing only ~1.2% fat and ~2.1% protein, but around 6–7% lactose [37]. These values are much lower in fat (and higher in lactose) than cow’s milk, reflecting a composition closer to human milk [38]. Earlier Soviet-era research in Kazakhstan and Russia documented similar profiles—e.g., ~2.3% protein and a rich vitamin C content in mare’s milk—but such studies often focused on single nutrients or specific herds [9,45]. What has been lacking is an integrated analysis combining all major components over lactation. Our data address this gap by providing a comprehensive compositional profile for Kazakh mare’s milk under natural pasture conditions, which was not previously available in the regional literature.

In Europe, interest in mare’s milk has grown for its nutritional and hypoallergenic value. Prior European studies (e.g., in Poland, France, and Italy) have typically examined specific aspects of composition or milk from specialized farms. Polish researchers, for example, reported an average fat content of around 1.1–1.2% in a managed horse herd [16]. This aligns closely with our findings and with values for Arabian mare’s milk (≈1.16% fat) reported by Bornaz et al. in Tunisia [46]. Notably, European studies show that mare’s milk fat can vary with lactation stage: one long-term Polish trial found fat ~1.5% in early lactation, dropping below 0.8% by late lactation [16]. Protein in European mare’s milk generally ranges from ~1.6 to 2.4%, also comparable to our Kazakh mare data [16,47,48]. Overall, European mare’s milk data confirm the same low-fat, high-lactose pattern, but these studies did not simultaneously consider as many factors (lactation stage, mare age, etc.) as our current study. By covering chemical composition and physical properties across different lactation periods, our work adds a new integrative perspective to the European findings.

Mare’s milk is also traditionally consumed by ethnic groups in Western China (e.g., Kazakhs in Xinjiang), mostly in fermented form. Chinese studies have primarily focused on fermentation processes and microbial properties of mare’s milk products rather than detailed raw milk composition. Nevertheless, the available reports indicate a composition consistent with other regions: high water and lactose content with modest fat and protein levels [49]. Xinjiang mare’s milk used for kumis production is described as having a lactose content on par with Mongolian mare’s milk (often around 6% or more) and fat under 2% [50]. Our study provides valuable new data in this context by documenting the full compositional range of fresh mare’s milk from Kazakhstan—information that can also be relevant to understanding Chinese and other Central Asian mare’s milk, where comprehensive compositional analyses have not been well represented in the literature.

Recent studies on Arabian horse milk (e.g., in North Africa) show similar compositional trends. In Tunisia, Hachana et al. examined raw milk from Arabian mares and found an average fat content of about 1.5–1.7%, with protein around 2%—again, markedly low fat levels, even slightly higher than in our Kazakh samples [51]. An earlier analysis of fermented Arabian mare’s milk also reported ~1.16% fat [46], essentially identical to European and Kazakh values. Intriguingly, breed differences can influence milk composition. For example, the Lipizzaner horse (studied in Slovenia) produced milk with an unusually high fat content (~4%) [16], whereas lighter horse breeds and those on natural pasture yield much lower fat. Aside from such exceptions, mare’s milk from Arabian or other breeds in arid regions still conforms to the general profile of high lactose, low fat, and easily digestible proteins [20]. Our findings on Kazakh mare’s milk fit squarely within this global context, while adding novel data from a region that had not been comprehensively profiled before.

Compared to these regional studies, our work is distinct in its scope. Many prior investigations targeted one class of nutrients (e.g., fatty acids in Europe, or fermentation quality in Asia) or looked at milk from intensive farms rather than free-grazing mares. In contrast, our study integrates multiple compositional parameters—measured in Kazakh mares over different lactation stages and ages. This integrated approach provides a holistic dataset that was previously lacking for Kazakh mare’s milk. By contextualizing our results against Mongolian, European, Chinese, and Arabian mare milk data, we underscore that our study adds new knowledge where little comprehensive data existed. In summary, the compositional values we report not only fall in line with those from other countries but also extend them by offering a more complete and contextualized picture. This comparative insight strengthens the novelty of our research, as it fills an important gap in the literature on mare’s milk composition in Central Asia.

The fatty acid composition of Kazakh mare’s milk observed in this study highlights a favorable lipid profile, notably characterized by a high proportion of PUFAs and a low content of SFAs, which collectively influence lipid health indices such as the AI and TI, depending on lactation stage and geographic region. In the present study, the AI and TI of Kazakh mare’s milk were found to range from 1.06 to 1.33 and 0.71 to 0.85, respectively, depending on lactation period, mare age, and foaling category. These values are slightly higher than previously reported ranges for mare’s milk—around 0.66 to 1.08, while TI ranges from about 0.29 to 0.56 [52], both of which are generally lower than values of other animals, indicating a more favorable fatty acid profile [53,54]. The dominant saturated fatty acids in the samples were palmitic acid (C16:0), lauric acid (C12:0), and myristic acid (C14:0), in agreement with an earlier study on milk [55]. The MUFA content, especially oleic acid (C18:1n9c), ranged from 18.28% to 20.96%, depending on location and physiological factors, consistent with data reported by Cais-Sokolińska et al. [16] in Polish cold-blooded mares. MUFAs are known to support cardiovascular health by improving the lipid profile, particularly when replacing saturated fats in the diet. The PUFA fraction, mainly linoleic acid (C18:2n6c) and alpha-linolenic acid (C18:3n3), contributed significantly to the favorable n6/n3 ratio, which ranged from 2.68 to 14.24. These ratios were better in the Zhambyl region and in summer-collected milk, aligning with recommendations suggesting an n6/n3 ratio below 5 for anti-inflammatory and cardioprotective effects [56].

Differences in FA composition and lipid quality indices across regions are more pronounced, largely reflecting diet (fresh grass vs. hay or concentrate) and geography. Mare’s milk fat is universally low in SFAs and relatively high in USFA than other animals, conferring a favorable atherogenicity profile [57]. In our Kazakh samples, the AI ranged 1.06–1.33 and the TI 0.71–0.85, values slightly above some literature reports for mares but still markedly better than those of ruminant milk [58]. For comparison, mare’s milk from a small Polish farm showed a mean AI of ~1.06 and TI ~0.68 [20], and prior studies note equine milk typically falls in the range AI ≈0.7–1.1 and TI ≈0.3–0.6—far lower than cow’s milk. Indeed, a recent experiment demonstrated that cow milk products have AI and TI values three- to six-fold higher than mare milk products [58]. The low SFA content in mare’s milk (palmitic, myristic, and lauric acids together are much lower than in bovine fat) and the abundance of anti-atherogenic unsaturated fats explain these differences. Our Kazakh mare’s milk had a lipid profile similar to those reported in Poland [20], with oleic acid (18:1 n-9) contributing ~18–21% of total FAs and substantial polyunsaturated fatty acids (PUFAs)—chiefly linoleic (18:2n-6) and α-linolenic acid (18:3n-3). However, the proportion of PUFAs and the omega-6/omega-3 ratio can vary widely with the feeding regime. Notably, Mongolian mares’ milk is renowned for its exceptionally high PUFA content under extensive grazing: one study found total PUFAs ~28–29% of milk fat, including an extraordinarily high α-linolenic acid level (~19% of FAs), and a very low n-6/n-3 ratio around 0.4 [59]. Such a profile is even more omega-3 rich than our Kazakh summer milk (which had ~18% PUFA and n-3 around 5–6%, yielding n-6/n-3 ratios in the range of ~3–5 in the best cases). In European mare’s milk, where some concentrate feeding may be used, PUFA percentages are often a bit lower (typically 15–20%) and n-6/n-3 ratios slightly higher (often 2–5%), but still nutritionally superior to bovine milk (which commonly has an n-6/n-3 ratio > 10). In our study, the n-6/n-3 ratio of Kazakh mare’s milk ranged from 2.7 (in lush summer pasture conditions) up to about 14 (in late autumn from older mares), illustrating how season and mare physiology can swing this health index. The Zhambyl region samples, from lower-altitude steppe, had particularly favorable ratios on par with Mongolian reports, presumably due to richer omega-3 forage. Overall, the literature confirms that mares managed on natural pasture—whether in Central Asia or Europe—produce milk fat with low AI and TI indices relative to other dairy species [58].

Seasonal, lactational, and regional factors together shape these differences and similarities. Summer grazing invariably boosts the omega-3 content (ALA) in mare’s milk, yielding lower AI and n-6/n-3 ratios, whereas in winter or off-pasture diets those metrics can worsen [59]. Early-lactation milk (and colostrum) tends to be richer in fat and protein, so geographic comparisons must consider sampling time postpartum [12]. The present Kazakh dataset is the first to comprehensively characterize mare’s milk composition under the traditional Central Asian pasture-based system and to directly compare it with diverse published data. Kazakhstan, along with Mongolia, is among the world’s top producers of mare’s milk, yet prior to our work there was a paucity of detailed information on the nutritional profile of Kazakh mares’ milk [48]. The novelty of our findings lies in capturing how local breed genetics and extensive management translate into milk composition that, while aligning with global norms for horse milk, exhibits enhanced nutritional indices under optimal conditions (e.g., summer grazing by younger mares). In summary, Kazakh mare’s milk demonstrates a rich amino acid spectrum and a PUFA-dominant fat profile comparable to mare’s milk from Mongolia, Europe, and Arab countries, with seasonal and regional nuances that reinforce its status as a uniquely wholesome dairy resource. These comparisons highlight that the cardioprotective lipid indices (low AI, low TI, and a balanced n-6/n-3 ratio) and the high-quality protein of mare’s milk are consistent advantages across equine milks, while our study adds new insight into the extent of variability due to environment and lactation—thereby filling an important gap in the mare’s milk literature and strengthening the case for its inclusion in functional foods and diets [48]. While lactation stage strongly influences mare’s milk lipid composition—with late lactation associated with peak PUFA content—donkey milk fat composition remains relatively consistent throughout the lactation cycle [60].

Among the EAAs, lysine, leucine, and valine were dominant. Lysine, an indispensable EAA required for growth and nitrogen balance, was particularly abundant (ranging from 0.45 to 0.71 mg/mL), consistent with values reported by Purkiewicz et al. [30] and Barłowska et al. [16] in Sokólski and Polish warmblood mares. Lysine is critical for protein synthesis and calcium absorption, making its presence especially important in milk intended for infants and individuals with protein malnutrition.

Leucine and valine, both branched-chain amino acids, also showed relatively high concentrations. These AAs are essential for muscle repair and energy metabolism, especially during growth or stress. The leucine content in our samples was higher in the early lactation period and in younger animals, aligning with previous observations that milk in early lactation has higher nutritional density [61].

In terms of NEAAs, glutamic acid, proline, and aspartic acid were predominant. Glutamic acid had the highest concentration overall (1.3–1.8 mg/mL), which supports its function as a precursor for the synthesis of gamma-aminobutyric acid, a key neurotransmitter involved in infant brain development. These values are comparable to those reported in previous studies of mare’s milk [12,62]. The elevated proline levels, particularly in mid-lactation and older mares, also indicate structural support for collagen synthesis and are essential for gut health and development in neonates.

Across studies, leucine, lysine, and arginine are consistently among the most abundant EAAs in equine milk, whereas methionine and histidine are relatively low. In our Kazakh samples, lysine was notably high (0.45–0.71 mg/mL), in line with values reported for Polish Sokólski and Arabian mares. The balance of total EAAs to non-essential AAs (NEAAs) in mare’s milk (approximately 1:1.2–1.4) is comparable between breeds, reflecting a protein composition optimized for foal growth and human nutrition. Nevertheless, environmental and management factors can modulate specific amino acid levels [12]. Seasonal and lactation-stage shifts have been documented: milk collected in mid-summer can show peak concentrations of certain AAs. For instance, in the highland pastures of Kyrgyzstan, proline, methionine, and tryptophan reached their highest levels in July milk, while valine peaked later in the summer [10]. Our findings in Kazakhstan mirror these trends—summer milk was enriched in glutamate, aspartate, serine, and histidine, whereas autumn milk contained more valine, leucine, methionine, and cysteine (likely due to progressive changes in pasture vegetation and lactation progression). Mare age and parity further influence amino acid profiles: Hachana et al. observed in purebred Arabian mares that as lactation advanced, protein content declined (with corresponding lower amino acids), although higher-parity mares produced slightly protein-richer milk early on [51]. We similarly noted that younger Kazakh mares (4–6 years) yielded milk with higher EAA concentrations than older mares, highlighting how physiological maturity affects milk protein quality. Overall, the amino acid composition of Kazakh mare’s milk aligns with the ranges reported in Mongolia, Europe, and elsewhere, but our dataset is novel in capturing how pasture-based seasonality and mare age interlink to shape these profiles.

Interestingly, methionine and cystine, both sulfur-containing amino acids, were present in moderate levels and varied significantly by region and foaling month. These amino acids play important roles in antioxidant defense via glutathione synthesis and are precursors for taurine, which is essential in neonatal development [63].

The results showed that essential amino acids comprised approximately 47–54% of the total amino acids in mare’s milk, aligning with the FAO/WHO/UNU recommendation for high-quality protein sources. Seasonal and physiological factors such as foaling number and mare age significantly influenced AA distribution. Older mares and those in later lactation showed a decrease in total EAAs, possibly due to physiological adaptation and reduced metabolic activity [64]. Compared to bovine milk, mare’s milk has a higher proportion of essential amino acids and a more balanced AA score, particularly in lysine, threonine, and histidine, which are often limiting in plant-based proteins [30,38].

A limitation of the present study is the absence of data on the hygienic quality of the mare’s milk, including microbiological indicators such as total bacterial count, coliform count, and somatic cell count. These parameters can influence certain physical, chemical, and biochemical properties of milk, and their omission may limit the interpretation of compositional variability. Future research will incorporate hygienic quality assessments alongside compositional profiling to provide a more integrated understanding of how microbiological status interacts with biological and environmental factors to shape milk quality.

## 5. Conclusions

This study provides the first integrated dataset on the physicochemical, amino acid, and fatty acid composition of Kazakh mare’s milk under pasture conditions, alongside key lipid health indices. Compared to data from other studies, Kazakh mare’s milk is characterized by a balanced essential amino acid profile, a favorable n-6/n-3 ratio, and low atherogenic and thrombogenic indices, supporting its value as a functional food for cardiovascular health. Seasonal and lactation-stage variations were evident, particularly in polyunsaturated fatty acids and essential amino acids, underlining the influence of pasture-based feeding and physiological stage on milk quality. One limitation of this work is the lack of data on the hygienic quality of the milk, which may influence certain compositional traits. Future studies will address this by integrating microbiological and hygienic assessments with compositional analysis, enabling a more complete understanding of the factors affecting mare’s milk quality and its potential for functional dairy product development. These results not only strengthen the nutritional profile of mare’s milk in the global context but also provide a scientific foundation for promoting Kazakh mare’s milk in functional dairy markets. Future studies should explore protein fraction-specific amino acid profiles and bioactive peptides to further clarify health benefits.

## Figures and Tables

**Table 1:** FAME retention times, linear parameters, LOD, and LOQ ratios for the FA standards.

#	Fatty Acid	Retention Time	R^2^	Calibration Curve Equation	Calibration Range (µg/mL)	LOD (µg/mL)	LOQ (µg/mL)
1	C4:0	7.111	0.9876	y = 0.0047x + 29.719	40.4–404	1.21	3.74
2	C6:0	8.96	0.9954	y = 0.0039x + 23.897	40.4–404	1.74	3.25
3	C8:0	11.492	0.9996	y = 0.0043x + 21.439	40.4–404	0.89	2.64
4	C10:0	15.256	0.9998	y = 0.0039x + 1.8014	40.8–408	0.61	2.83
5	C11:0	17.847	0.9998	y = 0.0038x − 0.1529	20.4–204	0.44	2.38
6	C12:0	19.458	0.9985	y = 0.0034x + 14.09	40.4–404	0.81	2.59
7	C13:0	22.432	0.9991	y = 0.0031x + 1.6595	20.3–203	0.86	2.68
8	C14:0	23.365	0.9985	y = 0.0042x + 15.744	40.4–404	1.64	3.94
9	C14:1	25.406	0.9880	y = 0.0042x + 12.928	20.4–204	1.28	2.94
10	C15:0	25.792	0.9452	y = 0.0023x + 0.1732	20.3–203	0.55	2.65
11	C15:1	26.514	0.9997	y = 0.0043x + 15.084	20.4–204	0.49	2.42
12	C16:0	26.94	0.9943	y = 0.0026x − 3.5892	61.2–612	1.44	2.41
13	C16:1	28.074	0.9971	y = 0.0033x + 10.884	20.4–204	1.62	2.85
14	C17:0	29.207	0.9748	y = 0.0027x + 1.9941	21.0–210	0.27	2.78
15	C17:1	30.018	0.9914	y = 0.0032x + 8.7811	20.4–204	0.35	2.94
16	C18:0	30.427	0.9860	y = 0.0026x + 10.246	40.8–408	0.46	2.36
17	C18:1n9t	31.047	0.9912	y = 0.0034x + 16.506	20.2–202	1.38	3.24
18	C18:1n9c	31.203	0.9973	y = 0.0024x + 22.074	40.4–404	0.44	2.46
19	C18:2n6t	32.527	0.9975	y = 0.0029x + 14.645	20.2–202	1.96	2.92
20	C18:2n6c	32.669	0.9959	y = 0.0029x + 20.107	20.2–202	1.15	3.45
21	C20:0	33.468	0.9987	y = 0.0027x + 18.09	40.8–408	0.37	2.08
22	C18:3n6c	34.048	0.9930	y = 0.0023x + 17.388	20.3–203	0.34	2.99
23	C20:1n9c	34.524	0.9933	y = 0.0031x + 8.5154	20.2–202	1.46	2.48
24	C18:3n3c	34.684	0.9393	y = 0.0023x + 20.688	20.4–204	0.78	2.34
25	C21:0	35.145	0.9986	y = 0.0024x + 10.468	20.3–203	1.16	3.42
26	C20:2	36.168	0.9994	y = 0.0035x + 4.2141	20.4–204	1.24	3.86
27	C22:0	36.845	0.9975	y = 0.0028x + 9.5838	40.5–405	1.16	2.46
28	C20:3	37.426	0.9948	y = 0.0026x + 6.2057	20.4–204	1.15	2.58
29	C22:1	38.039	0.9518	y = 0.0029x + 12.565	20.4–204	0.28	2.84
30	C20:3	38.169	0.9981	y = 0.0026x + 18.341	20.4–204	0.57	2.78
31	C23:0	38.467	0.9817	y = 0.0029x + 14.205	20.3–203	1.44	2.56
32	C20:4	38.917	0.9947	y = 0.0025x + 11.377	20.2–202	1.14	2.68
33	C22:2	40.031	0.9981	y = 0.0038x + 14.392	20.4–204	1.26	2.98
34	C24:0	41.09	0.9998	y = 0.0024x + 39.734	40.4–404	0.54	2.76
35	C20:5	41.403	0.9994	y = 0.0064x + 29.666	20.4–204	0.72	2.26
36	C24:1	42.207	0.9961	y = 0.0034x + 41.385	20.4–204	1.55	2.78
37	C22:6	45.503	0.9995	y = 0.0092x + 34.921	20.3–203	0.56	2.68

R—correlation coefficient; LOD—limit of detection; LOQ—limit of quantification.

**Table 2:** AA retention times, linear parameters, LOD, and LOQ ratios for the AA standards.

#	Amino Acid	Retention Time	R^2^	Calibration Curve Equation	Calibration Range (µg/mL)	LOD (µg/mL)	LOQ (µg/mL)
1	Aspartic acid	8.387	0.9996	y = 1429.4x − 1421.1	1–100	0.06	0.19
2	Glutamic acid	9.486	0.9998	y = 2015.3x − 1076.4	1–100	0.03	0.10
3	Serine	10.417	0.9979	y = 3084.3x − 9055.9	1–100	0.15	0.46
4	Asparagine	10.427	0.9977	y = 1816.8x + 4740.6	1–100	0.16	0.47
5	Histidine	11.803	0.9997	y = 2305.8x − 2791.1	1–100	0.06	0.17
6	Arginine	12.596	0.9997	y = 1170.1x − 1494.1	1–100	0.06	0.18
7	Threonine	12.712	0.9998	y = 2538.2x − 3107.3	1–100	0.04	0.13
8	Alanine	13.224	0.9989	y = 3568.1x + 4727	1–100	0.05	0.14
9	Proline	13.589	0.9995	y = 7214.1x + 1226.3	1–100	0.07	0.21
10	Cysteine	14.640	0.9997	y = 8793.4x + 1234.3	1–100	0.04	0.11
11	Tyrosine	16.117	0.9997	y = 1904.6x + 1495.3	1–100	0.10	0.32
12	Valine	17.124	0.9991	y = 1858.6x + 2927.6	1–100	0.06	0.17
13	Methionine	17.331	0.9985	y = 1812.4x + 294.6	1–100	0.13	0.38
14	Cystine	17.842	0.9995	y = 783.1x + 1292.6	1–100	0.07	0.22
15	Isoleucine	19.284	0.9995	y = 3035.1x + 2905.7	1–100	0.07	0.22
16	Leucine	19.463	0.9985	y = 1717.1x + 447.11	1–100	0.13	0.39
17	Phenylalanine	20.076	0.9996	y = 1852.5x + 2184.7	1–100	0.06	0.20
18	Lysine	20.983	0.9996	y = 2401.7x + 3659.9	1–100	0.07	0.20

R—correlation coefficient; LOD—limit of detection; LOQ—limit of quantification.

**Table 3:** Chemical composition of Almaty and Zhambyl regions, Kazakh mare milk during lactation.

Parameter	Region		Lactation Period		Foaling Number			Foaling Month				Mare Age			*p*-Value by				
	Almaty (*n* = 120)	Zhambyl (*n* = 120)	Summer (*n* = 96)	Autumn (*n* = 144)	1 (*n* = 20)	2–4 (*n* = 130)	5 or More (*n* = 90)	April (*n* = 60)	May (*n* = 70)	June (*n* = 80)	July (*n* = 30)	4–6 Years (n = 80)	7–9 Years (n = 76)	10 Years over (n = 84)	Region	Month	Foaling Number	Foaling Month	Mare Age
Fat (%)	0.85 ± 0.39	1.29 ± 0.37	0.71 ± 0.27	1.31 ± 0.38	0.95 ± 0.21	1.09 ± 0.26	1.07 ± 0.16	0.96 ± 0.11	0.97 ± 0.29	1.05 ± 0.10	1.36 ± 0.15	1.09 ± 0.19	1.06 ± 0.28	1.06 ± 1.20	0.0001	0.0001	0.4781	0.0160	0.6632
Protein (%)	1.71 ± 0.18	1.58 ± 0.33	1.62 ± 0.16	1.66 ± 0.18	1.67 ± 0.19	1.63 ± 0.16	1.61 ± 0.18	1.68 ± 0.13	1.66 ± 0.11	1.60 ± 0.17	1.58 ± 0.21	1.63 ± 0.15	1.64 ± 0.17	1.65 ± 0.19	0.0001	0.2535	0.5717	0.0453	0.2790
Casein (%)	1.20 ± 0.28	1.21 ± 0.24	1.26 ± 0.14	1.04 ± 0.18	1.24 ± 0.26	1.21 ± 0.26	1.20 ± 0.27	1.24 ± 0.27	1.22 ± 0.23	1.18 ± 0.29	1.16 ± 0.27	1.22 ± 0.26	1.18 ± 0.27	1.22 ± 0.27	0.8648	0.0001	0.6519	0.0294	0.4478
Lactose (%)	6.41 ± 0.56	6.47 ± 0.33	6.51 ± 0.32	6.39 ± 0.53	6.48 ± 0.24	6.42 ± 0.54	6.45 ± 0.37	6.41 ± 0.69	6.51 ± 0.25	6.45 ± 0.43	6.40 ± 0.33	6.42 ± 0.46	6.44 ± 0.56	6.45 ± 0.36	0.4979	0.1588	0.7136	0.1302	0.7905
Urea (mg/dL)	46.39 ± 3.51	31.64 ± 1.96	32.46 ± 1.16	43.39 ± 3.34	38.65 ± 7.43	38.55 ± 2.87	39.79 ± 3.49	36.56 ± 4.05	42.88 ± 4.53	39.29 ± 3.39	34.22 ± 5.30	38.53 ± 3.53	37.85 ± 3.86	40.53 ± 3.65	0.0004	0.0106	0.7848	0.2291	0.6087
Citric acid (%)	0.05 ± 0.01	0.10 ± 0.02	0.07 ± 0.00	0.07 ± 0.01	0.08 ± 0.02	0.07 ± 0.00	0.06 ± 0.01	0.07 ± 0.01	0.07 ± 0.01	0.08 ± 0.01	0.08 ± 0.01	0.07 ± 0.01	0.07 ± 0.01	0.08 ± 0.01	0.0003	0.8023	0.6016	0.8090	0.0886
Acidity °Dornic (°D)	9.31 ± 1.71	8.43 ± 1.13	10.24 ± 0.39	6.82 ± 0.07	8.46 ± 0.71	8.87 ± 0.31	8.97 ± 0.58	8.87 ± 0.48	8.85 ± 0.39	8.58 ± 0.34	9.66 ± 1.61	8.68 ± 0.39	8.98 ± 0.41	8.95 ± 0.62	0.1177	0.0001	0.6386	0.2562	0.6710
SNF (%)	8.79 ± 0.64	9.19 ± 1.01	8.66 ± 0.34	9.20 ± 0.89	9.11 ± 0.72	8.98 ± 0.93	8.98 ± 0.82	8.88 ± 1.14	9.08 ± 0.69	8.99 ± 0.79	8.98 ± 0.88	9.00 ± 0.93	9.00 ± 1.00	8.97 ± 0.69	0.0114	0.0008	0.6871	0.3723	0.8881
TS (%)	9.09 ± 0.67	9.03 ± 1.07	8.86 ± 0.47	9.19 ± 1.06	9.02 ± 1.01	9.06 ± 0.88	9.07 ± 0.90	8.83 ± 0.86	9.11 ± 0.85	9.17 ± 0.88	9.12 ± 1.05	9.05 ± 0.90	8.97 ± 0.91	9.16 ± 0.87	0.7089	0.0447	0.8693	0.1114	0.3560

Values are presented as the mean ± SD. SNF, solids-not-fat; TS, total solids.

**Table 4:** Effect of Region, Lactation Period, Foaling Number, Foaling Month, and Mare Age on the Fatty Acid Composition (% of Total Fatty Acids) of Kazakh Mares’ Milk.

Parameter	Region		Lactation Period		Foaling Number			Foaling Month				Mare Age			*p*-Value by				
	Almaty (*n* = 120)	Zhambyl (*n* = 120)	Summer (*n* = 96)	Autumn (*n* = 144)	1 (*n* = 20)	2–4 (*n* = 130)	5 or More (*n* = 90)	April (*n* = 60)	May (*n* = 70)	June (*n* = 80)	July (*n* = 30)	4–6 Years (n = 80)	7–9 Years (n = 76)	10 Years over (n = 84)	Region	Month	Foaling Number	Foaling Month	Mare Age
C4:0	0.14 ± 0.03	0.16 ± 0.02	0.04 ± 0.01	0.21 ± 0.03	0.13 ± 0.05	0.14 ± 0.03	0.15 ± 0.02	0.13 ± 0.04	0.09 ± 0.01	0.15 ± 0.03	0.29 ± 0.14	0.13 ± 0.03	0.18 ± 0.05	0.13 ± 0.02	0.5842	0.0004	0.8097	0.0130	0.4269
C6:0	0.15 ± 0.01	0.13 ± 0.01	0.18 ± 0.01	0.07 ± 0.01	0.14 ± 0.03	0.15 ± 0.03	0.14 ± 0.01	0.13 ± 0.01	0.14 ± 0.03	0.15 ± 0.02	0.16 ± 0.03	0.14 ± 0.01	0.15 ± 0.02	0.14 ± 0.01	0.2563	0.0001	0.8859	0.4813	0.6190
C8:0	2.32 ± 0.80	1.88 ± 0.81	1.70 ± 0.79	2.41 ± 0.73	2.04 ± 0.84	2.19 ± 0.87	2.09 ± 0.74	2.25 ± 0.74	2.17 ± 0.90	2.09 ± 0.84	1.97 ± 0.84	2.12 ± 0.91	2.25 ± 0.82	2.05 ± 0.76	0.0079	0.0001	0.6571	0.3440	0.3171
C10:0	6.18 ± 1.66	4.46 ± 1.11	5.08 ± 1.56	5.71 ± 1.72	5.66 ± 1.37	5.56 ± 1.85	5.29 ± 1.48	5.85 ± 1.31	5.67 ± 1.74	5.37 ± 1.78	4.28 ± 1.81	5.46 ± 1.97	5.71 ± 1.52	5.27 ± 1.56	0.0001	0.0462	0.5919	0.0056	0.2854
C11:0	1.30 ± 0.11	0.20 ± 0.09	0.96 ± 0.13	0.77 ± 0.12	0.66 ± 0.29	0.95 ± 0.15	0.72 ± 0.14	0.83 ± 0.19	0.92 ± 0.16	0.86 ± 0.17	0.66 ± 0.30	0.85 ± 0.17	0.90 ± 0.18	0.79 ± 0.14	0.0001	0.3140	0.4493	0.4429	0.6153
C12:0	7.74 ± 2.06	6.21 ± 1.83	7.02 ± 2.34	7.17 ± 1.96	7.41 ± 1.34	7.27 ± 2.37	6.82 ± 1.78	7.61 ± 1.50	7.38 ± 2.21	6.94 ± 2.24	5.69 ± 2.42	7.19 ± 2.62	7.42 ± 1.71	6.77 ± 1.90	0.0002	0.7321	0.0455	0.0070	0.2067
C14:0	8.01 ± 1.96	7.10 ± 1.88	7.86 ± 2.19	7.50 ± 1.83	7.95 ± 1.45	7.72 ± 2.26	7.45 ± 1.59	8.01 ± 1.47	7.97 ± 1.64	7.57 ± 2.27	6.07 ± 2.38	7.72 ± 2.45	7.81 ± 1.71	7.39 ± 172	0.0202	0.3721	0.5396	0.0035	0.3846
C14:1	0.63 ± 0.04	0.74 ± 0.06	0.44 ± 0.05	0.82 ± 0.04	0.69 ± 0.15	0.65 ± 0.05	0.70 ± 0.05	0.62 ± 0.06	0.65 ± 0.07	0.69 ± 0.06	0.80 ± 0.13	0.65 ± 0.07	0.69 ± 0.06	0.67 ± 0.06	0.1456	0.0001	0.5305	0.1578	0.6571
C16:0	22.19 ± 3.30	21.87 ± 4.49	24.02 ± 3.25	20.84 ± 3.65	22.91 ± 2.95	21.70 ± 4.34	22.43 ± 3.05	22.64 ± 3.10	22.59 ± 3.10	22.25 ± 3.17	18.85 ± 3.60	21.64 ± 4.73	21.99 ± 3.55	22.5 ± 3.11	0.6793	0.0001	0.4323	0.0031	0.0359
C16:1	6.55 ± 1.99	8.59 ± 1.98	7.76 ± 2.11	7.16 ± 2.27	7.68 ± 2.21	7.56 ± 2.39	7.24 ± 2.13	7.15 ± 1.76	7.60 ± 1.93	7.46 ± 2.61	7.15 ± 2.88	7.21 ± 2.21	7.42 ± 2.03	7.53 ± 2.44	0.0001	0.1899	0.6186	0.5478	0.5506
C17:1	0.52 ± 0.16	0.65 ± 0.23	0.51 ± 0.16	0.61 ± 0.22	0.62 ± 0.18	0.59 ± 0.18	0.55 ± 0.21	0.58 ± 0.19	0.56 ± 0.19	0.55 ± 0.21	0.52 ± 0.19	0.54 ± 0.23	0.58 ± 0.18	0.59 ± 0.19	0.0010	0.0172	0.4197	0.3124	0.4062
C18:0	1.10 ± 0.15	0.87 ± 0.13	1.63 ± 0.16	0.62 ± 0.12	0.85 ± 0.12	0.90 ± 0.28	1.11 ± 0.17	1.14 ± 0.28	1.00 ± 0.16	0.92 ± 0.16	0.89 ± 0.32	1.07 ± 0.19	1.05 ± 0.23	0.89 ± 0.13	0.2833	0.0001	0.0249	0.045	0.5088
C18:1n9c	18.28 ± 3.79	20.96 ± 4.84	19.78 ± 4.88	19.13 ± 4.15	18.69 ± 3.37	19.48 ± 4.73	20.40 ± 4.01	19.99 ± 4.67	19.36 ± 2.64	18.29 ± 4.78	18.01 ± 5.54	18.56 ± 5.53	19.04 ± 3.60	20.44 ± 4.13	0.0022	0.4810	0.0472	0.1947	0.0378
C18:2n6c	17.53 ± 4.37	12.27 ± 4.32	15.22 ± 3.23	15.58 ± 4.12	15.13 ± 2.39	15.35 ± 5.67	15.43 ± 4.18	15.69 ± 4.82	15.91 ± 5.33	14.77 ± 3.96	14.74 ± 5.39	14.61 ± 5.33	15.61 ± 4.87	15.83 ± 5.02	0.0001	0.7319	0.8836	0.4974	0.0321
C18:3n3c	2.37 ± 0.30	3.82 ± 0.61	5.71 ± 0.47	1.27 ± 0.22	2.79 ± 0.39	2.84 ± 1.10	3.26 ± 0.57	2.57 ± 0.52	3.05 ± 0.59	3.07 ± 0.52	3.33 ± 1.16	2.67 ± 0.53	2.77 ± 0.49	3.42 ± 0.59	0.0220	0.0001	0.0484	0.0306	0.0338
C18:3n6c	0.87 ± 0.14	2.59 ± 0.43	0.07 ± 0.01	2.51 ± 0.29	1.36 ± 0.36	1.40 ± 0.27	1.89 ± 0.39	1.64 ± 0.44	1.62 ± 0.38	1.43 ± 0.36	1.32 ± 0.68	1.36 ± 0.34	1.65 ± 0.39	1.71 ± 0.39	0.0001	0.0001	0.0454	0.6951	0.0163
SFA	49.13	42.88	48.49	45.3	47.75	46.58	46.2	48.59	47.93	46.3	38.86	46.32	47.46	45.93					
MUFA	25.98	30.94	28.49	27.72	27.68	28.28	28.89	28.34	28.17	26.99	26.48	26.96	27.73	29.23					
PUFA	20.77	18.68	21	19.36	19.28	19.59	20.58	19.9	20.58	19.27	19.39	18.64	20.03	20.96					
n3	2.37	3.82	5.71	1.27	2.79	2.84	3.26	2.57	3.05	3.07	3.33	2.67	2.77	3.42					
n6	18.4	14.86	15.29	18.09	16.49	16.75	17.32	17.33	17.53	16.2	16.06	15.97	17.26	17.54					
n6/n3	7.76	3.89	2.68	14.24	5.91	5.9	5.31	6.74	5.75	5.28	4.82	5.98	6.23	5.13					
AI	1.33	1.14	1.26	1.23	1.32	1.25	1.19	1.29	1.29	1.27	1.06	1.31	1.27	1.17					
TI	0.84	0.78	0.80	0.71	0.87	0.82	0.81	0.85	0.85	0.85	0.72	0.86	0.83	0.79					

Values are presented as the mean ± SD. Values are expressed as means (%) of total fatty acids. SFA—saturated fatty acids; MUFA—monounsaturated fatty acids; PUFA—polyunsaturated fatty acids; n-3 = omega-3 fatty acids; n-6 = omega-6 fatty acids; n-6/n-3 = ratio of omega-6 to omega-3 fatty acids; AI—atherogenic index; TI—thrombogenic index.

**Table 5:** Effect of Region, Lactation Period, Foaling Number, Foaling Month, and Mare Age on the Amino Acid Content (mg/mL) of Kazakh Mare’s Milk.

Parameter	Region		Lactation Period		Foaling Number			Foaling Month				Mare Age			*p*-Value by				
	Almaty (*n* = 120)	Zhambyl (*n* = 120)	Summer (*n* = 96)	Autumn (*n* = 144)	1 (*n* = 20)	2–4 (*n* = 130)	5 or More (*n* = 90)	April (*n* = 60)	May (*n* = 70)	June (*n* = 80)	July (*n* = 30)	4–6 Years (n = 80)	7–9 Years (n = 76)	10 Years over (n = 84)	Region	Month	Foaling Number	Foaling Month	Mare Age
Aspartic acid	1.35 ± 0.09	1.86 ± 0.14	1.49 ± 0.11	1.82 ± 0.11	0.78 ± 0.12	1.34 ± 0.11	2.15 ± 0.16	0.97 ± 0.13	1.33 ± 0.15	1.59 ± 0.16	1.85 ± 0.23	0.99 ± 0.11	1.57 ± 0.15	2.16 ± 0.16	0.1543	0.0210	0.0484	0.0361	0.0165
Glutamic acid	3.06 ± 0.35	4.85 ± 0.40	1.51 ± 0.27	5.58 ± 0.36	3.49 ± 0.27	3.91 ± 0.34	4.64 ± 0.45	3.37 ± 0.31	4.59 ± 0.42	3.77 ± 0.38	4.12 ± 0.42	3.50 ± 0.33	3.98 ± 3.46	4.37 ± 4.62	0.0103	0.0001	0.0125	0.2091	0.0316
Serine	0.73 ± 0.07	1.26 ± 0.17	0.89 ± 0.11	1.06 ± 0.15	0.60 ± 0.06	1.03 ± 0.14	1.03 ± 0.13	0.63 ± 0.05	1.03 ± 0.14	1.03 ± 0.14	1.58 ± 0.22	0.64 ± 0.06	1.34 ± 0.17	1.02 ± 0.15	0.0334	0.4958	0.3624	0.0296	0.0234
Asparagine	0.24 ± 0.03	0.25 ± 0.04	0.06 ± 0.01	0.37 ± 0.04	0.26 ± 0.03	0.22 ± 0.03	0.27 ± 0.04	0.27 ± 0.03	0.26 ± 0.03	0.24 ± 0.03	0.17 ± 0.02	0.25 ± 0.03	0.23 ± 0.03	0.25 ± 0.04	0.9708	0.0001	0.5240	0.3505	0.7868
Histidine	0.23 ± 0.02	0.28 ± 0.02	0.12 ± 0.01	0.35 ± 0.01	0.21 ± 0.02	0.25 ± 0.02	0.28 ± 0.02	0.22 ± 0.01	0.26 ± 0.02	0.27 ± 0.02	0.28 ± 0.02	0.21 ± 0.01	0.28 ± 0.02	0.28 ± 0.02	0.1810	0.0001	0.2950	0.2761	0.1053
Arginine	1.06 ± 0.11	1.20 ± 0.12	0.95 ± 0.14	1.89 ± 0.09	1.21 ± 0.11	1.02 ± 0.11	1.28 ± 0.12	1.31 ± 0.12	1.18 ± 0.13	1.06 ± 0.10	0.73 ± 0.09	1.07 ± 0.10	1.12 ± 0.11	1.21 ± 0.13	0.5246	0.0001	0.2594	0.0112	0.5955
Threonine	0.95 ± 0.02	0.96 ± 0.03	1.25 ± 0.37	0.43 ± 0.07	0.41 ± 0.06	0.51 ± 0.02	0.65 ± 0.03	0.06 ± 0.01	0.49 ± 0.02	0.69 ± 0.02	1.11 ± 0.38	0.05 ± 0.01	0.68 ± 0.03	0.85 ± 0.03	0.0350	0.0074	0.0496	0.0019	0.0150
Alanine	0.16 ± 0.01	0.12 ± 0.01	0.22 ± 0.01	0.04 ± 0.00	0.13 ± 0.01	0.15 ± 0.02	0.13 ± 0.01	0.16 ± 0.02	0.14 ± 0.01	0.13 ± 0.01	0.12 ± 0.01	0.14 ± 0.01	0.16 ± 0.01	0.12 ± 0.01	0.1579	0.0001	0.5696	0.0469	0.2348
Proline	0.45 ± 0.02	0.47 ± 0.03	0.58 ± 0.03	0.28 ± 0.02	0.45 ± 0.02	0.46 ± 0.03	0.47 ± 0.03	0.42 ± 0.02	0.47 ± 0.03	0.48 ± 0.03	0.51 ± 0.03	0.43 ± 0.02	0.46 ± 0.03	0.50 ± 0.03	0.7638	0.0001	0.8882	0.3009	0.3417
Cysteine	0.42 ± 0.04	0.45 ± 0.05	0.58 ± 0.04	0.22 ± 0.03	0.26 ± 0.03	0.40 ± 0.03	0.52 ± 0.05	0.35 ± 0.04	0.41 ± 0.04	0.49 ± 0.04	0.51 ± 0.03	0.34 ± 0.03	0.47 ± 0.05	0.48 ± 0.04	0.7211	0.0001	0.1053	0.2706	0.1451
Tyrosine	0.11 ± 0.01	0.12 ± 0.01	0.04 ± 0.00	0.16 ± 0.01	0.10 ± 0.01	0.11 ± 0.01	0.12 ± 0.01	0.09 ± 0.00	0.21 ± 0.01	0.12 ± 0.01	0.12 ± 0.00	0.10 ± 0.01	0.11 ± 0.01	0.12 ± 0.01	0.7145	0.0001	0.6512	0.2038	0.3873
Valine	1.78 ± 0.13	1.65 ± 0.13	0.76 ± 0.01	2.36 ± 0.12	1.64 ± 0.13	1.73 ± 0.14	1.72 ± 0.13	1.41 ± 0.10	1.81 ± 0.14	1.85 ± 0.14	1.79 ± 0.15	1.52 ± 0.13	1.77 ± 0.13	1.87 ± 0.14	0.6044	0.0001	0.8496	0.1893	0.2419
Methionine	0.11 ± 0.01	0.15 ± 0.01	0.19 ± 0.01	0.05 ± 0.00	0.12 ± 0.01	0.13 ± 0.01	0.14 ± 0.01	0.11 ± 0.01	0.12 ± 0.01	0.15 ± 0.02	0.15 ± 0.01	0.12 ± 0.01	0.14 ± 0.01	0.14 ± 0.02	0.0422	0.0001	0.5893	0.0328	0.5410
Cystine	0.37 ± 0.03	0.47 ± 0.03	0.61 ± 0.03	0.13 ± 0.02	0.37 ± 0.02	0.41 ± 0.03	0.45 ± 0.03	0.35 ± 0.02	0.43 ± 0.03	0.44 ± 0.02	0.48 ± 0.03	0.37 ± 0.03	0.45 ± 0.03	0.42 ± 0.03	0.1161	0.0001	0.0476	0.0248	0.2691
Isoleucine	1.43 ± 0.14	2.24 ± 0.22	2.73 ± 0.21	0.49 ± 0.06	1.59 ± 0.17	1.68 ± 0.19	2.10 ± 0.22	1.37 ± 0.15	1.83 ± 0.20	2.00 ± 0.21	2.29 ± 0.24	1.51 ± 0.17	1.95 ± 0.20	2.03 ± 0.22	0.0262	0.0001	0.0472	0.0150	0.0291
Leucine	0.12 ± 0.22	0.12 ± 0.19	0.08 ± 0.12	0.13 ± 0.19	0.08 ± 0.15	0.12 ± 0.12	0.12 ± 0.21	0.12 ± 0.03	0.12 ± 0.12	0.12 ± 0.22	0.12 ± 0.18	0.12 ± 0.21	0.13 ± 0.21	0.12 ± 0.21	0.7058	0.0001	0.4291	0.6709	0.5027
Phenylalanine	0.17 ± 0.02	0.20 ± 0.02	0.05 ± 0.01	0.28 ± 0.02	0.18 ± 0.01	0.17 ± 0.02	0.21 ± 0.02	0.16 ± 0.01	0.19 ± 0.02	0.19 ± 0.01	0.19 ± 0.01	0.15 ± 0.01	0.20 ± 0.01	0.20 ± 0.02	0.2832	0.0001	0.2386	0.4094	0.2223
Lysine	0.82 ± 0.06	1.03 ± 0.09	0.04 ± 0.01	1.27 ± 0.07	0.72 ± 0.06	0.95 ± 0.08	0.93 ± 0.07	0.79 ± 0.06	0.86 ± 0.07	0.99 ± 0.08	1.17 ± 0.10	0.86 ± 0.07	0.94 ± 0.08	0.98 ± 0.08	0.0152	0.0001	0.4060	0.0153	0.0251
EAA	5.61	6.63	5.22	5.36	4.95	5.54	6.15	4.24	5.68	6.26	7.1	4.54	6.09	6.47					
NEAA	7.95	11.05	6.93	11.55	7.65	9.05	11.06	7.92	9.35	10.05	10.19	7.83	9.89	10.65					

Values are expressed as means ± standard deviations in milligrams per milliliter (mg/mL) of mare milk. EAA—essential amino acids; NEAA—non-essential amino acids.

## Data Availability

The original contributions presented in this study are included in the article. Further inquiries can be directed to the corresponding author.

## Abbreviations

The following abbreviations are used in this manuscript:

SFA	saturated fatty acids
PUFA	polyunsaturated fatty acids
MUFA	monounsaturated fatty acids
USFA	unsaturated fatty acids
AI	atherogenic index
TI	thrombogenic index
SNF	solids-not-fat
TS	total solids
FAME	fatty acid methyl esters
GC	gas chromatography
FID	flame ionization detector
AA	Amino acid
FA	Fatty acid
HPLC	high-performance liquid chromatography
PITC	phenyl isothiocyanate
EAA	Essential amino acids
NEAA	non-essential amino acids
PCA	Principal component analysis
LOD	limits of detection
LOQ	Limits of quantification
